# Investigating ultrastructural morphology in MIRAGE syndrome (SAMD9)-derived fibroblasts using transmission electron microscopy.

**DOI:** 10.12688/f1000research.129559.1

**Published:** 2023-02-10

**Authors:** Federica Buonocore, Monika Balys, Glenn Anderson, John C. Achermann

**Affiliations:** 1 Genetics and Genomic Medicine Research and Teaching Department, UCL Great Ormond Street Institute of Child Health, University College London, London, UK; 2 Histopathology Department, Great Ormond Street Hospital for Children NHS Foundation Trust, London, UK

**Keywords:** SAMD9, endosomes, MIRAGE syndrome, transmission electron microscopy.

## Abstract

**Background**: Heterozygous *de novo* variants in the gene *SAMD9* cause the complex multisystem disorder, MIRAGE syndrome. Patients are characterised by myelodysplasia, infections, growth restriction, adrenal insufficiency, gonadal dysfunction and enteropathies. Pathogenic variants in SAMD9 are gain-of-function and enhance its role as a growth repressor, leading to growth restriction of many tissues. Two studies have reported changes in skin fibroblasts derived from MIRAGE patients, more specifically identifying enlarged endosomes. We have also previously shown subtle changes in endosome size in patients’ fibroblasts compared to controls. However, these variations in endosomes were not as marked as those described in the literature.

**Methods**: We have performed an observational study using transmission electron microscopy (EM) in a larger number of cells derived from three patients’ fibroblasts to assess ultrastructure morphology compared to control images.

**Results**: Consistent changes were observed in cell organelles in all patient samples. In particular, increased endosomal activity was detected, characterised by augmented pinocytosis and vesicle budding, increased endosome number, as well as by large lysosomes and endosomes. Endoplasmic reticulum was also prominent. Mitochondria appeared enlarged in selected cells, possibly due to cellular stress. Cell nuclei did not display major differences compared to controls.

**Conclusions**: EM is a powerful tool to investigate morphological features of tissues and cell organelles, although EM data could be affected by sample preparation methodology, therefore potentially explaining the variability between independent studies, and its analysis can be dependent on the experience of the researcher. The increased endosomal activity we have observed in patients’ fibroblasts could indicate that SAMD9 regulates endocytosis of receptors, acting as an endosome fusion facilitator, or in lysosomal activation. However, the precise mechanism(s) by which SAMD9 regulates cell growth is still not fully understood, and further studies are needed to elucidate its pathogenic pathway and develop therapeutic approaches to support patients.

## Introduction

MIRAGE syndrome (myelodysplasia, infection, restriction of growth, adrenal hypoplasia, genital (gonadal) phenotypes, and enteropathy) (OMIM: 617053) is a well-established complex multisystem disorder caused by pathogenic gain-of-function variants in the gene *SAMD9*. ^1^^,^^2^ Changes in this gene were first described in 2016 and to date more than 100 affected individuals have been reported. We have recently undertaken a meta-analysis of all published *SAMD9*-associated variants and shown that the range of clinical phenotypes is very variable. ^3^ Increasingly, children with MIRAGE syndrome are diagnosed who do not have adrenal insufficiency, and a large proportion of children and young people with *SAMD9* variants present with myelodysplastic syndrome (MDS) alone. ^3^^,^^4^

SAMD9 has been extensively shown to be a growth repressor in *in vitro* cellular models, explaining the typical phenotypic growth restriction and tissue hypoplasia observed in children with this condition. However, the molecular mechanism or mechanisms by which SAMD9 affects cell growth and proliferation are still unknown. Two studies have reported characteristic enlarged endosomes in skin fibroblasts derived from MIRAGE patients, including “giant” endosomes in some cells. ^1^^,^^5^ These authors proposed that endosome dysfunction results in reduced recycling of epidermal growth factor receptor (EGFR), with consequent decreased cell growth and proliferation. ^1^ We have also observed somewhat larger vesicles in our patients’ fibroblasts using transmission electron microscopy (EM) imaging and Rab5a and Rab7a as early- and late- endosomal markers respectively, in live fibroblast cultures. ^2^ However, these EM studies were only reviewed at low power and in limited sections, and more detailed high-power imaging was not undertaken.

EM remains an extremely powerful approach for visualising and analysing intracellular structures in biological samples. This technique is extensively used in clinical diagnosis as well as research settings, and therefore represents an invaluable analytical tool. In this study, we have used EM to systematically analyse ultrastructural morphology of skin fibroblasts, which had been previously derived from MIRAGE patients, ^2^ to investigate any potential characteristic features that could help elucidate the molecular role of SAMD9.

## Methods

### Samples

The clinical phenotypes and initial EM findings have been previously described in our original report of MIRAGE syndrome. ^2^ In brief, these eight patients were all delivered preterm with fetal growth restriction and needing intensive care. Additional features included primary adrenal insufficiency, recurrent viral and bacterial infections, persistent diarrhoea, and bone marrow dysfunction. Fibroblasts from three of the eight patients were obtained: patient 1 with a c.1376G>A, p.R459Q change; patient 2 with a c.2054G>A, p.R685Q change; patient 3 with c.2948T>G, p.I983S. Patient 3 was also found to harbour a secondary somatic mosaic c.2294delA, p.N765Tfs*13 change in haematopoietic cells. All three patients had a 46,XY karyotype.

### Ethics/Ethical statement

Written informed consent of the patients’ parents was obtained for research and diagnosis prior to inclusion in the study (NRES London-Bloomsbury 07/Q0508/24).

### Fibroblasts

Skin fibroblasts from three patients and from one control (46,XY) were grown in Dulbecco’s Modified Eagle Medium (DMEM) supplemented with 10% FBS and 1% penicillin/streptomycin at 37°C in a humidified atmosphere (5% CO _2_). All fibroblast cultures used were negative for mycoplasma contamination.

### Transmission electron microscopy

Fibroblasts were detached from culture flasks by trypsin digestion and centrifuged at 3000 rpm for 15 minutes to form a pellet for EM processing. After removal of the medium, all cell pellets were fixed in 2.5% glutaraldehyde in 0.1 M sodium cacodylate buffer followed by secondary fixation in 1% osmium tetroxide. Samples were dehydrated in graded ethanol, transferred to a transitional fluid (propylene oxide) and then infiltrated and embedded in Agar 100 epoxy resin. Polymerisation was at 60 ° C for 48 hours. Ultrathin sections (90 nm) were cut using a Diatome diamond knife on a Leica Ultracut UC7 ultramicrotome (Leica Microsystems, Germany). Sections were picked up on Athene 300 mesh copper grids and stained with 70% alcoholic uranyl acetate and Reynold’s lead citrate for contrast. Sections were then examined using a JEOL 1400 transmission electron microscope (JEOL, Japan) and images recorded using an AMT XR80 digital camera (Advanced Microscopy Techniques, US). Independent images from randomly selected control or patient fibroblasts were studied. A total of 100 fibroblasts were examined from each culture, initially at x1,000 magnification. Representative cells showing particular ultrastructural features were recorded at higher magnifications for further assessment. Interpretation of EM changes was made by comparing patient-derived samples with the control sample that was processed in parallel, as well as based on the extensive experience of EM analysis of one author (G. A.).

## Results

All three patient samples showed similar ultrastructural features which were distinct from the control sample. Given the qualitative, observational nature of EM, the major consistent changes are described below.

**Figure 1.**
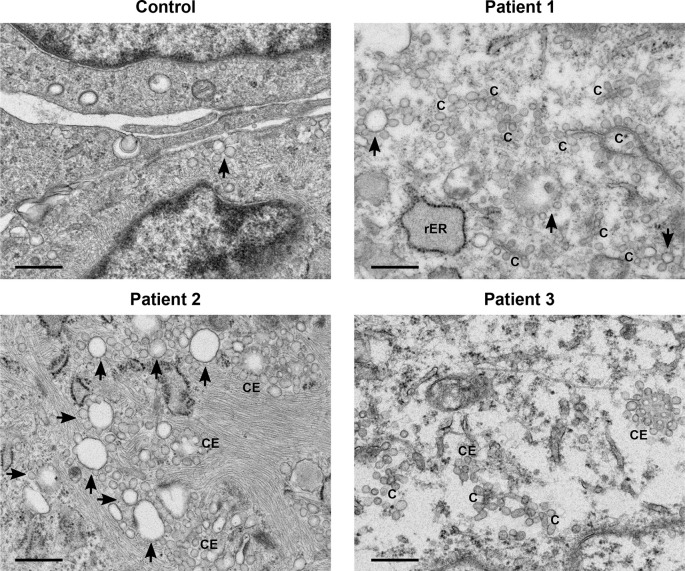
Increased endosomal activity was clear in all patients’ cells. All three patients’ samples displayed features of increased endosomal activity. These included increased number of pinocytic vesicles, many endosome chains (C) and budding (indicated by arrows), as well as clusters of endosomes (CE). Scale bars 500 nm.

**Figure 2.**
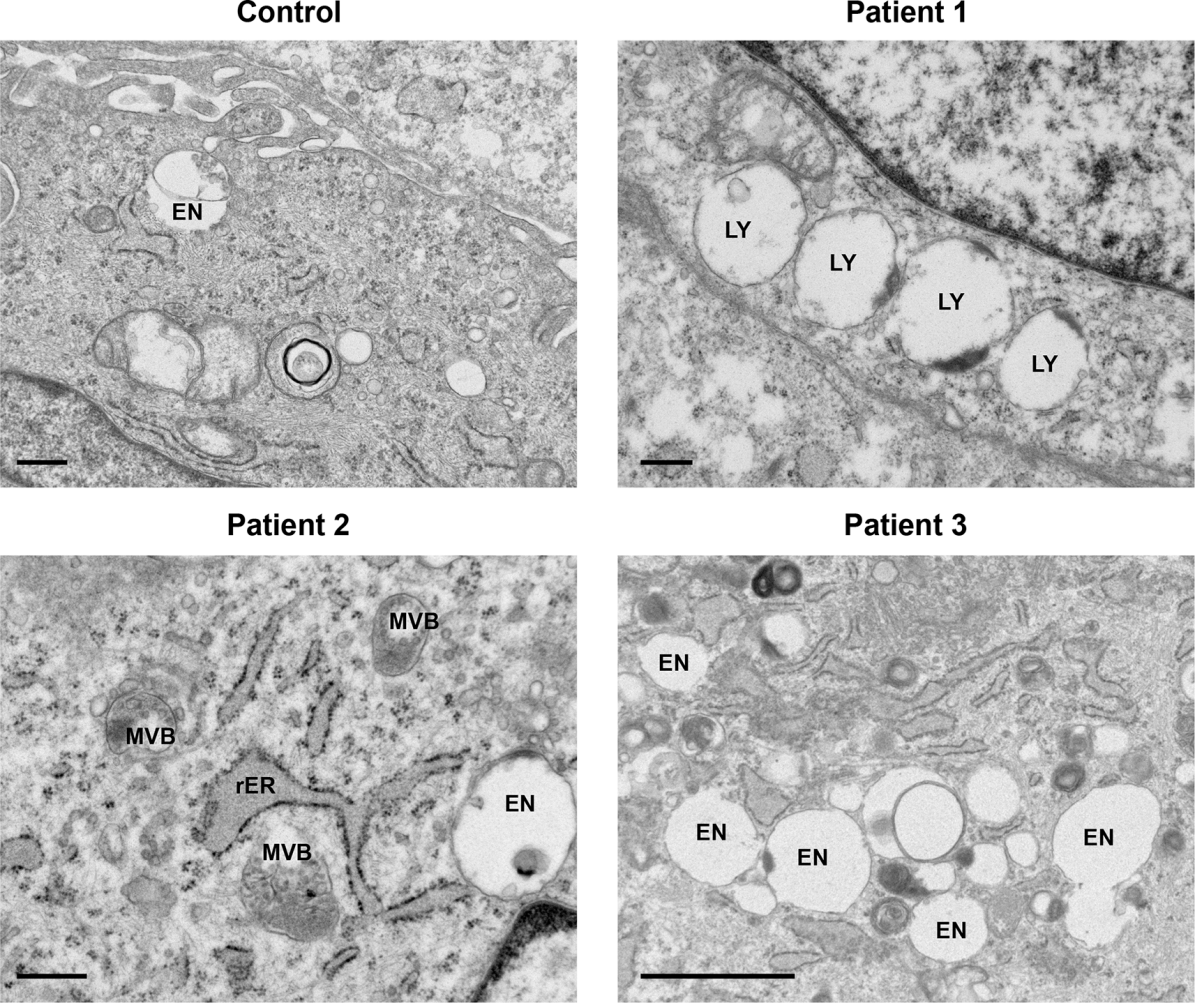
Late endosomes and lysosomes displayed characteristics of increased endosomal activity. Increased endosomal activity was also demonstrated by the presence of large, mainly empty lysosomes (LY) with dense debris localised to the border; enlarged empty endosomes (EN) and late endosomes filled with multivesicular bodies (MVB). Rough endoplasmic reticulum (rER). Control, Patient 1 and Patient 2 scale bars 500 nm; Patient 3 scale bar 2 μm.

A general increase in endosome number and activity was observed in the patients’ fibroblasts ( Figure 1). Characteristic features of increased endosomal activity included pinocytic vesicles, early endosomes showing chains and clumps of vesicles, as well as vesicle budding ( Figure 1). Several late endosomes had multivesicular bodies (MVB) ( Figure 2). Evidence of increased endosomal activity was also demonstrated by enlarged empty endosomes and large (over 1.0 μm diameter), single membrane bound lysosomes, which were mainly empty with a rim of electron dense material ( Figure 2).

**Figure 3.**
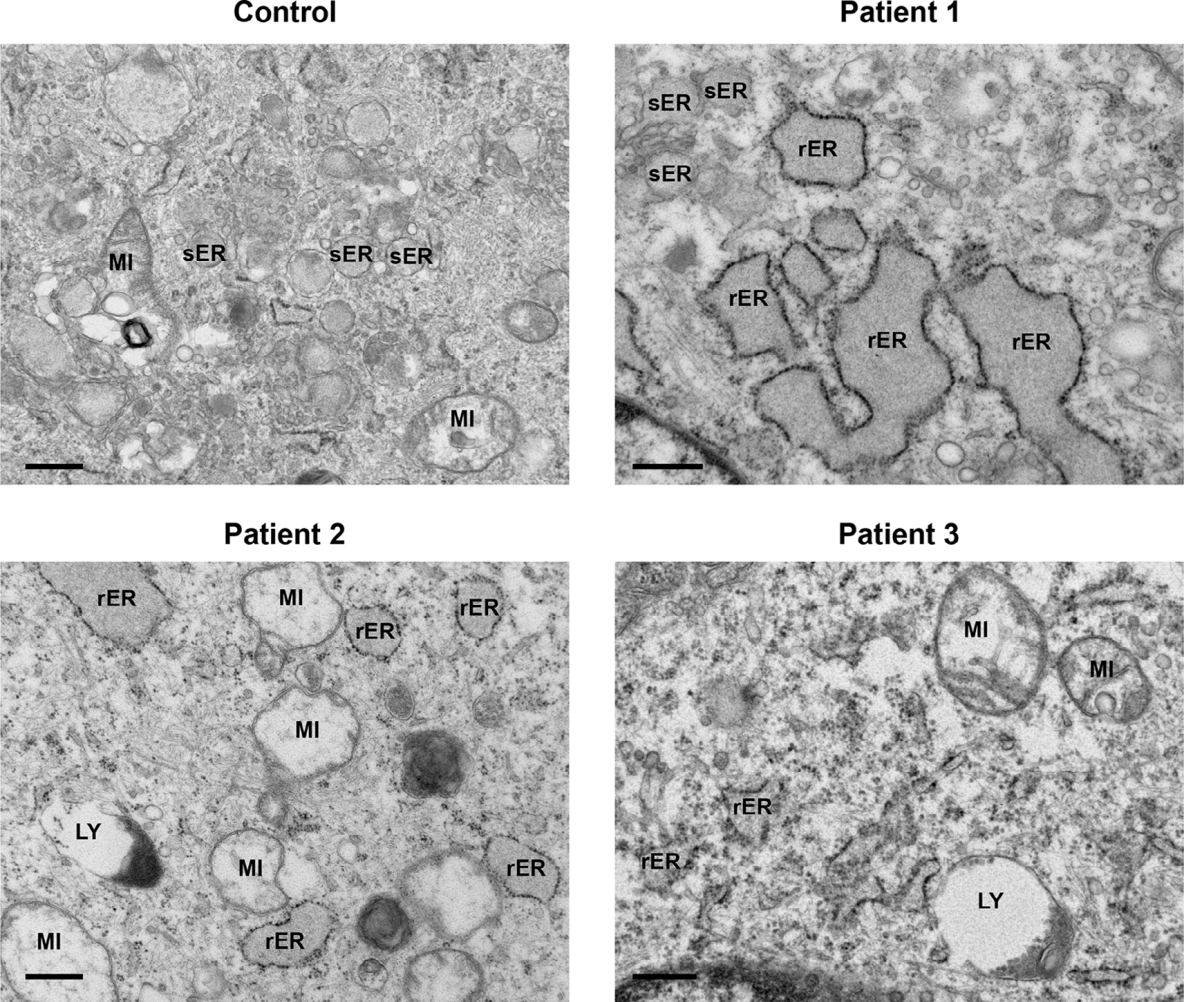
Mitochondria and endoplasmic reticulum presented dilated. Both control and patients’ cells displayed a regular array of organelles. Often patients’ mitochondria (MI) were expanded. Smooth endoplasmic reticulum (sER) appeared dilated and was filled with granular material (Patient 1), but without any ribosomes lining the membrane. Rough endoplasmic reticulum (rER) was full of granular electron dense material, and a moderate distention of the cisternae was also observed. Lysosomes (LY). Scale bars 500 nm.

All patient-derived cells displayed a regular array of organelles ( Figure 3). Mitochondria did not show any significant pathological features except swelling and internal cristae disruption in a few cells. Endoplasmic reticulum was prominent in several fibroblasts and was mainly of the rough endoplasmic type (rER). A moderate distention of the cisternae was observed, with granular electron dense material. Dilatation of smooth endoplasmic reticulum (sER) was also present, indicated by an irregular outline with granular material within, but without any obvious ribosomes lining the membranes ( Figure 3, Patient 1, top left).

**Figure 4.**
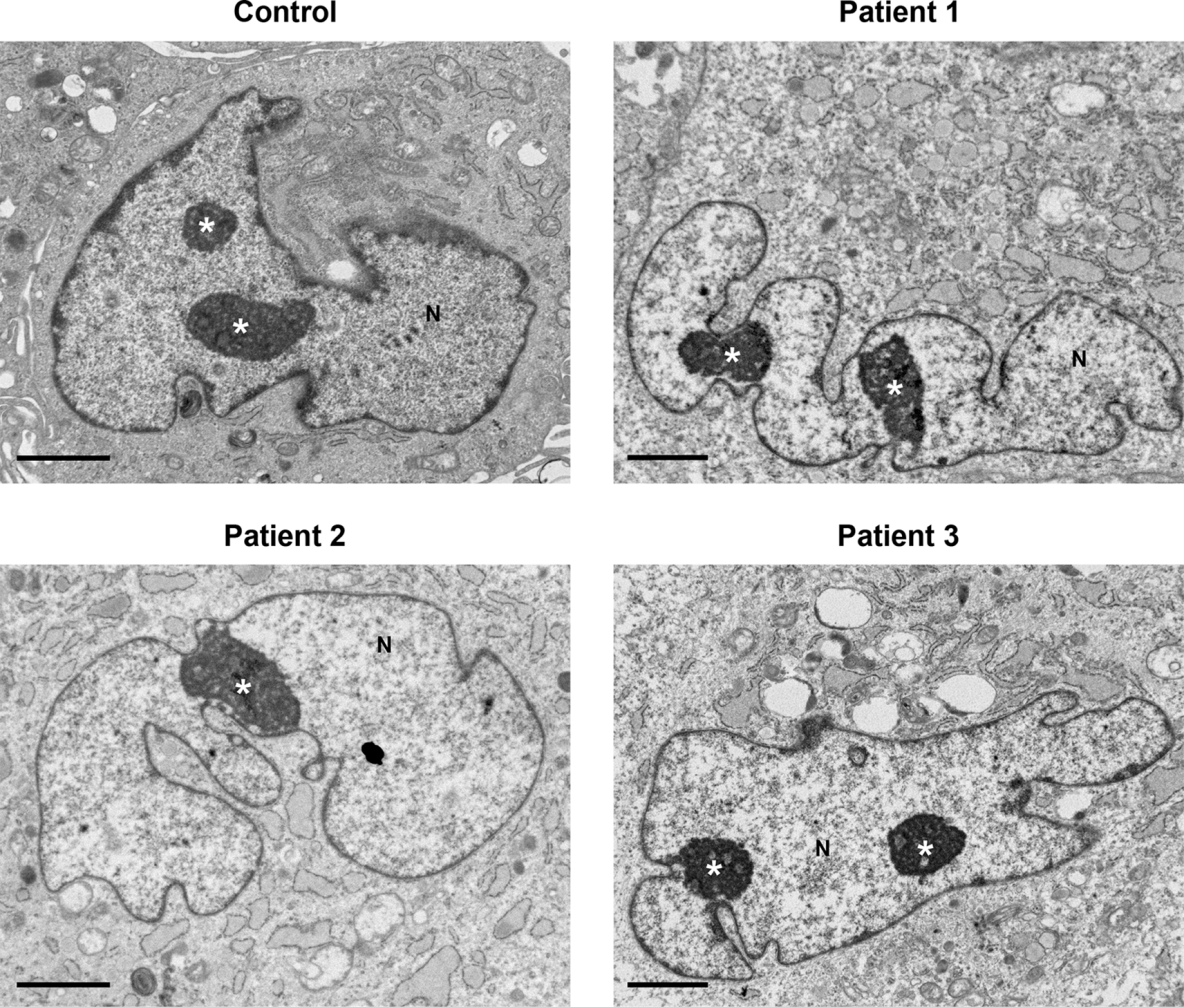
Nuclei and nucleoli did not display any differences between control and patients’ cells. Nuclei (N) had an irregular nuclear envelope, with a marginated chromatin pattern in both control and patients’ cells. Nucleoli (indicated by the white asterisk *) were pronounced in size and in various cells multiple nucleoli were seen. Several cytoplasmic features in patients’ cells (described in Figures 1– 3) are also seen at lower power here. Scale bars 2 μm.

No major differences in cell nuclei between control and patient samples were observed ( Figure 4). Several features were common in both control and patient cells, including an irregular nuclear envelope with a marginated chromatin pattern, nucleoli often prominent in size and occasionally multiple nucleoli present in one cell.

## Discussion

In this study, we have used EM to examine ultrastructural morphology of fibroblasts derived from MIRAGE patients. Although our early specimens were variable, ^2^ we have now obtained and systematically analysed more images from our original fibroblast samples. All samples were well preserved with ample numbers of cells for analysis. The three patients’ samples showed essentially similar features and were distinct from the control sample, demonstrating that carrying *SAMD9* variants resulted in differences in intracellular structures.

Evidence of increased endosomal activity was present in all patients’ samples. This was reflected by an increase in pinocytosis and budding in early endosomes, and the presence of large endosomes and lysosomes. Endosomes can be broadly classified as early endosomes, late endosomes and recycling endosomes, which will fuse with lysosomes for degradation of waste matter. Lysosomes are single membrane bound organelles filled with hydrolytic enzymes capable of degrading many types of biomolecules, cellular organelles and micro-organisms. ^6^ Thus, lysosomes typically appear as sphere-shaped sacks with electron dense contents. Taken together, consistent markers of increased endosome activity in patients’ samples were seen.

We also observed a dilation of both rER and sER. The main function of rER is the synthesis and modification of proteins that need to be delivered to organelles within the cell or secreted from the cell. sER is associated with the synthesis of lipids such as cholesterol and phospholipids, which are essential for the formation of cellular membranes. sER also plays a role in glycogen metabolism. ^7^ Glycogen content and intermediate filaments were at varying levels in all samples and no excess lipid was detected.

Mitochondria were easily detected in EM images, as they are bounded by a double membrane and the inner membrane forms the characteristic infolding of lamellar cristae and matrix space. Their major role is the provision of energy by the production of ATP and phosphorylation of ADP to regulate cellular metabolism. ^8^ We detected bloated mitochondria in a few cells; however, this is quite a common feature and could indicate a sign of cellular stress, such as a delay in fibroblast preservation.

Cell nuclei did not show any major differences between control and patients’ samples and appeared with an irregular nuclear envelope. Irregularity of nuclear membrane provides an increased area of contact between the nucleus and the cytoplasm and may suggest heightened metabolic activity. ^9^

EM is widely used in clinical settings to diagnose specific conditions, such as lysosomal storage disorders, glomerular diseases and metabolic and congenital myopathies, ^10^^–^^12^ however, over the past years its use has been valuable in research settings too. Indeed, EM has been used to further investigate the detailed structures of cell organelles to validate potential novel findings discovered through research studies. For example, EM has been used to: a) assess ciliary ultrastructural defects in ciliary disorders ^13^; b) identify stored material in lysosomal storage disorders, including neuronal ceroid lipofuscinoses and Batten disease ^14^; and c) isolate highly infectious and contagious microorganisms, primarily viruses, to enable production of vaccines in microbial diseases, e.g. SARS-CoV-2/coronavirus. ^15^ However, there is no standard protocol for EM and different preparation methods can be used, which can often affect the observed results.

The most widely used method is “traditional ultrathin-section EM”, ^16^ which in brief consists of a series of steps (fixation, wash, dehydration, embedding) to preserve the specimen before sectioning and staining. This has been proven to maintain cellular integrity and sample structure, without leading to cellular artefacts. This approach is, therefore, potentially more reliable. In our original study, cultured fibroblasts were first pelleted and then fixed in 2.5% glutaraldehyde followed by 1% osmium tetroxide. They were then processed into agar 100 resin, sectioned (90 nm) and stained with uranyl acetate and lead citrate. In contrast, in other studies reporting giant endosomes in MIRAGE patients, ^1^^,^^5^ fibroblasts were seeded on a chamber slide and then fixed with 2.5% glutaraldehyde followed by 1.0% osmium tetroxide. They were then embedded in Epon, sectioned (70 nm) and stained with uranyl acetate and lead citrate. The use of different methodologies, especially the initial step of cell pelleting compared to growing cells directly on chamber slides, might therefore account for some of the variability in the appearance of the vesicles and endosomes in different, independent studies.

Whilst SAMD9-associated conditions provide a fascinating model for the dynamic evolution of genetic disease, the exact mechanism by which SAMD9 regulates cell growth and mediates a cellular effect is yet to be fully established. The paralogous gene *SAMD9L* has been shown to regulate endosomal fusion through degradation of EGFRs. ^17^ Several patients with MIRAGE syndrome had characteristic enlarged endosomes, ^1^^,^^5^ therefore *SAMD9* could potentially be involved in the regulation of endocytosis of receptors acting as an endosome fusion facilitator, ^1^ or in lysosomal activation. ^18^ In our original study, we showed some large vesicles in our patient fibroblasts, but these were quite heterogenous. We also carried out live cell imaging of early and late endosomes and the changes in early endosomes from live imaging were only subtle. ^2^ In the present study, all three MIRAGE patients’ fibroblasts exhibited increased endosomal activity compared to controls, as well as large lysosomes. These data could, therefore, still support the role of SAMD9 in recycling of EGFRs.

SAMD9 has also been associated with the viral host defence mechanism, especially poxviruses, ^19^^–^^21^ and tumour suppression. ^17^^,^^22^ More recently, it has been shown to have nucleic acid binding capacity, important for antiviral and antiproliferative functions. ^23^ As more insight is obtained on the biological function or functions of SAMD9, detailed data from patient-derived materials, and associated cellular ultrastructural changes, could provide more evidence for underlying disease mechanisms.

This study has several limitations. EM is a powerful tool, however its assessment remains subjective, as it mainly relies on the experience of the individual analysing the images. Qualitative evaluation can be difficult since the cells are in a three dimensions and EM is allowing visualisation of a very thin slice through the cell. The number of individuals with MIRAGE syndrome studied to date is relatively small, so data are limited.

In conclusion, we have observed evidence of increased endosomal activity in patients-derived fibroblasts carrying *SAMD9* variants compared to controls. These findings provide more data for ultrastructural morphology of SAMD9-associated conditions and supports the original findings of enlarged vesicles in patients’ fibroblasts. However, more insight into the pathogenic mechanisms of SAMD9 disruption is needed. Indeed, induced pluripotent stem cell lines (iPSC) have been generated from fibroblasts from two MIRAGE patients. ^2^^,^^24^ These represent a potentially important resource to further investigate the disease mechanisms of MIRAGE syndrome and model the underlying molecular basis, at least *in vitro.* Understanding the molecular function of SAMD9 is extremely important to help us develop personalised management and effective therapies for individuals with SAMD9-associated variants.

## Data Availability

OSF: Investigating ultrastructural morphology in MIRAGE syndrome (SAMD9)-derived fibroblasts using transmission electron microscopy. https://doi.org/10.17605/OSF.IO/Z8WVE. ^25^ This project contains the following extended data:
• Control (17.TIF files) • Patient1 (19.TIF files) • Patient2 (19.TIF files) • Patient3 (21.TIF files) Data are available under the terms of the Creative Commons BY (CC-BY) 4.0.
