# Supplementary material for: Investigating ultrastructural morphology in MIRAGE syndrome-derived fibroblasts using transmission electron microscopy
Source: F1000Res. 2024 Feb 13;12:155. Originally published 2023 Feb 10. [Version 2] doi: 10.12688/f1000research.129559.2 (PMC10904937; doi:10.12688/f1000research.129559.2)
Supplement: Peer review history

# Reviewer report: Reviewer response for version 2

**DOI:** 10.5256/f1000research.162387.r246555

**Published:** 2024-02-28

**Authors:** Satoshi Narumi

**Affiliations:** 1 Department of Molecular Endocrinology, National Center for Child Health and Development, Tokyo, Japan

The manuscript is well written. I have no further comments on it.

Is the work clearly and accurately presented and does it cite the current literature?

Yes

If applicable, is the statistical analysis and its interpretation appropriate?

Not applicable

Are all the source data underlying the results available to ensure full reproducibility?

Yes

Is the study design appropriate and is the work technically sound?

Yes

Are the conclusions drawn adequately supported by the results?

Yes

Are sufficient details of methods and analysis provided to allow replication by others?

Yes

Reviewer Expertise:

molecular genetics; pediatric endocrinology

I confirm that I have read this submission and believe that I have an appropriate level of expertise to confirm that it is of an acceptable scientific standard.

# Reviewer report: Reviewer response for version 1

**DOI:** 10.5256/f1000research.142249.r162986

**Published:** 2023-06-19

**Authors:** Sushree Sangita Sahoo

**Affiliations:** 1 Department of Hematology, St. Jude Children's Research Hospital, Memphis, TN, USA

Buonocore and colleagues present a research article titled "Investigating ultrastructural morphology in MIRAGE syndrome (SAMD9)-derived fibroblasts using transmission electron microscopy". The authors have provided a detailed description of SAMD9 patient fibroblast ultrastructure using TEM. In addition, the authors have outlined all specific cellular organelles with major or minor changes, and concluded that SAMD9 patient fibroblasts have increased endosomal activity. The paper's contents are interesting and will be helpful for researchers to focus on the outlined cell organelles to understand the physiological relevance of SAMD9 mutations. However, I have a major concern that the authors do not provide any statistical tests to delineate the mutant-specific cellular differences, especially for their increased endosomal conclusion.

Is the work clearly and accurately presented and does it cite the current literature?

Yes

If applicable, is the statistical analysis and its interpretation appropriate?

Yes

Are all the source data underlying the results available to ensure full reproducibility?

Yes

Is the study design appropriate and is the work technically sound?

Yes

Are the conclusions drawn adequately supported by the results?

Yes

Are sufficient details of methods and analysis provided to allow replication by others?

Yes

Reviewer Expertise:

bone marrow failure, myelodysplastic syndromes, HSC biology, experimental hematology

I confirm that I have read this submission and believe that I have an appropriate level of expertise to confirm that it is of an acceptable scientific standard, however I have significant reservations, as outlined above.

# Reviewer report: Reviewer response for version 1

**DOI:** 10.5256/f1000research.142249.r162985

**Published:** 2023-02-27

**Authors:** Timothy S Olson

**Affiliations:** 1 Cell Therapy and Transplant Section, Division of Oncology, Children's Hospital of Philadelphia, Philadelphia, PA, USA

Buonocore and colleagues present a reanalysis of Electron Microscopy samples derived from primary fibroblasts of 3 patients with MIRAGE syndrome originally published in their 2017 J Clin Invest report. In this prior report they had concluded that the endosomal abnormalities identified in MIRAGE fibroblasts by other investigators (Narumi, 2016, etc.) were not a consistent feature in their samples. However, using different prep techniques and a co-author with extensive EM analysis experience, they now conclude that Narumi and colleagues were correct in identifying increased endosomal activity and number, as well as large late endosome/lysosome structure as a characteristic feature of MIRAGE.  

 Comments:

1. This study presents a reanalysis of EM studies that have been previously published, and essentially refute the interpretation of these studies that was published in 2017. While I think this update is indeed critical to provide, since it does not involve analysis of new samples or by new techniques and is rather a reinterpretation of the previously published EM studies, this reviewer wonders whether these updated data would be better to include as an erratum to the original study, rather than as an independent report. If the authors feel differently, they should comment as to why these data represent sufficient originality that they should be considered as an independent report. 
2. Have the authors studied EM appearance of fibroblasts from patients with SAMD9/SAMD9L mutations who lack syndromic features of MIRAGE? Do such samples still show endosomal abnormalities, or are those abnormalities characteristic only of a MIRAGE phenotype? What about GoF versus LoF mutations, both of which are associated with MDS, though at distinct ages?
3. No source of the control fibroblasts is provided. Healthy individual? Age-matched? Cell line? How can the control cell have an irregular nuclear envelope (page 4) if it is in theory a normal cell? Is the irregularity artefactual based on the EM prep? 
4. In the discussion, the authors suggest that the EM prep used in the original study was to fix the fibroblasts after pelleted and before placement on a slide, whereas other investigators have plated live fibroblasts on slides first and then fixed. I'm confused as to which technique was used in the current study, and whether difference in technique can explain why the current study confirms the work of Narumi et al., whereas the previous 2017 work from these investigators saw only mild endosomal abnormalities.     

Is the work clearly and accurately presented and does it cite the current literature?

Yes

If applicable, is the statistical analysis and its interpretation appropriate?

Not applicable

Are all the source data underlying the results available to ensure full reproducibility?

Yes

Is the study design appropriate and is the work technically sound?

Yes

Are the conclusions drawn adequately supported by the results?

Yes

Are sufficient details of methods and analysis provided to allow replication by others?

Yes

Reviewer Expertise:

MIRAGE Syndrome.  Pediatric MDS Predisposition Syndromes.  Bone Marrow Failure

I confirm that I have read this submission and believe that I have an appropriate level of expertise to confirm that it is of an acceptable scientific standard, however I have significant reservations, as outlined above.

# Reviewer report: Reviewer response for version 1

**DOI:** 10.5256/f1000research.142249.r162984

**Published:** 2023-02-16

**Authors:** Satoshi Narumi

**Affiliations:** 1 Department of Molecular Endocrinology, National Center for Child Health and Development, Tokyo, Japan

In this article by Federica Buonocore and colleagues, skin fibroblasts established from three patients with MIRAGE syndrome (with distinct SAMD9 variants) were analyzed by TEM with a standardized protocol. The authors described the presence of disease-specific features, including ones previously reported and the others (increased pinocytosis and budding in early endosomes) that are novel. These observations suggest that the endosome-lysosome system is not only morphologically but also functionally abnormal. The extended data of this article contain a large number of images, which is highly appreciated as a very useful resource for researchers.

 Minor comments

1. (SAMD9) in the title necessary? I think all three patients had typical MIRAGE syndrome.
2. Patient 1 in this paper is the same person as Patient 4 in JCI 2017? Patient 2 is this paper is the same person as Patient 6 in JCI 2017? 
  Cells derived from Patient 3 are unreported?
3. It would be easier to understand if you clarify the relevance of JCI 2017 and this paper in the Methods section. Did you thaw the frozen stored cells and re-prepare the TEM samples? Or is this a re-observation of a previously prepared TEM samples that were in storage?
4. All results presented in this study are qualitative. If possible, I think objectivity in the features would be increased if there is something that can be evaluated quantitatively, such as the number and size of organelles per field of view. (If it is difficult, I think it is acceptable to leave it as it is now)

Is the work clearly and accurately presented and does it cite the current literature?

Yes

If applicable, is the statistical analysis and its interpretation appropriate?

Not applicable

Are all the source data underlying the results available to ensure full reproducibility?

Yes

Is the study design appropriate and is the work technically sound?

Yes

Are the conclusions drawn adequately supported by the results?

Yes

Are sufficient details of methods and analysis provided to allow replication by others?

Yes

Reviewer Expertise:

molecular genetics; pediatric endocrinology

I confirm that I have read this submission and believe that I have an appropriate level of expertise to confirm that it is of an acceptable scientific standard, however I have significant reservations, as outlined above.
